# Evaluation of the activity of antimicrobial peptides against bacterial vaginosis

**DOI:** 10.1515/biol-2022-0927

**Published:** 2024-07-30

**Authors:** Xuning Kang, Ting Zhao, Yuzhu Song, Jinyang Zhang, Tao Yuan, Qinqin Han

**Affiliations:** College of Life Science and Technology & Affiliated Hospital, Kunming University of Science and Technology, Kunming, 650500, Yunnan, China

**Keywords:** antimicrobial peptides, gram-positive bacteria, gram-negative bacteria

## Abstract

New drugs for the treatment of bacterial vaginosis (BV) are yet to be developed due to concerns that they may contribute to the increase in antibiotic resistance in BV. Antimicrobial peptides (AMPs) are one of the most promising options for next-generation antibiotics. In this study, we investigated the bacteriostatic activity of the AMPs Pexiganan, plectasin, melittin, and cathelicidin-DM against Gram-negative and Gram-positive bacteria both *in vitro* and in a mouse model of BV infection. The results showed that Pexiganan, melittin, and cathelicidin-DM had significant antibacterial activity against both Gram-negative and Gram-positive bacteria. AMPs have great potential for clinical application in the treatment of vaginitis, and this study provides an experimental basis for their use in the active immunoprophylaxis of BV.

## 1 Introduction

Bacterial vaginosis (BV) is a common mucosal infection that is frequently contracted by some women and usually difficult to cure in patients with the gynecological disease known as vaginitis [1,2], which is characterized by the presence of grayish-white, thin, and foul-smelling discharge [3]. BV is a disease characterized by the partial loss of intrinsic vaginal lactobacilli and the overgrowth of vaginal epithelial anaerobes [4]. The overall prevalence of BV among North American women of childbearing age is 27.4%, with a higher prevalence among black (33.2%) and Hispanic women (30.7%) compared to white (22.7%) or Asian women (11.1%) [5]. The most common form of vaginitis is bacterial, which is considered a “vaginal ecological disaster” caused by a variety of bacteria [6]. BV is the most common vaginal infection among women of childbearing age and can cause considerable physical and psychological discomfort [7]. BV is associated with serious public health consequences, including pelvic inflammatory disease [8], endometritis, postoperative infection, the acquisition and transmission of human immunodeficiency virus, and several adverse outcomes of prematurity and pregnancy [9]. Current evidence suggests that biofilm formation is an important mechanism underlying the transition of normal vaginal flora to those that cause BV [10]. During the pathogenesis of BV, changes occur in the levels of vaginal hydrogen peroxide (H_2_O_2_) and *Lactobacillus* bacteria [11]. Namely, the number of *Lactobacillus* species decreases, while the number of several pathogenic bacteria increases, especially that of anaerobes [12]. Biofilm recurrence occurs in roughly 40% of BV patients, and this phenomenon is thought to be caused not by newly acquired infection [13], but by the reanimation of biochemically inactivated biofilms [14]. Although some antibiotics are useful at inhibiting biofilm activity and have exhibited significant clinical results, none can eradicate the BV biofilm in all patients [15], suggesting that biofilm formation is only one of several mechanisms that underlie the pathogenesis of BV [16]. Currently, recommended treatment regimens for BV include the oral administration of multiple doses of metronidazole, tinidazole, or clindamycin [17], as well as the intravaginal administration of multiple doses of metronidazole or clindamycin [18], but they have a relatively short duration of efficacy and are prone to resistance [19]. Therefore, developing novel alternative therapeutic approaches and discovering new pharmacological targets is important for the prevention and treatment of recurrent BV.

Antimicrobial peptides (AMPs) are produced by multicellular organisms as a defense mechanism against competing pathogenic microbes [20]. They comprise a key component of an organism’s innate immune system. The mechanism of their action is not yet fully understood [21]. The generally accepted view is that AMPs and microorganisms [22], through either charge-based interactions or by receptor-mediated contact, result in AMP accumulation in the cell membrane and conformational changes [22], thereby disrupting the integrity of the membrane structure and resulting in the efflux of the cell contents during cell rupture [23]. Some naturally occurring AMPs, such as porcine proteins, exhibit strong antimicrobial activity, but in general, the activity of AMPs is enhanced by increasing their concentration in the vicinity of phagocytic granules, intestinal crypts, and degranulating phagocytes [24]. There may also be several intracellular antimicrobial mechanisms involved, such as the inhibition of nucleic acid synthesis, interference of protein synthesis, the inhibition of cell membrane synthesis, and so on [25]. The diversity and effectiveness of AMPs have also rendered them highly suitable for the development of antibacterial drugs [26]. Numerous studies have shown that AMPs have a variety of biological activities, such as antibacterial, antiviral, and wound healing promotion [27]. Studies of synergism that involve the co-administration of traditional antibiotics and AMPs as well as antibiotic-AMP conjugates [28], where an antibiotic is covalently bound to the peptide, have shown in several instances that these resistant strains can be targeted and killed [29]. In addition, synergistic effects arise from the combination of AMPs and antibiotics, while AMPs are less susceptible to causing bacterial and fungal resistance mutations when compared to conventional antibiotics [30]. The diversity and efficacy of AMPs have led to the development of new antimicrobial drugs and dozens of AMPs are currently being evaluated in clinical trials [31]. Currently, more than 3,100 AMPs have been described from a variety of plant and animal sources [32]. Pexiganan source from magainin is a potent and broad antimicrobial peptide spectrum with oral activity [33]. Pexiganan has been more and more studied and reported as effective antimicrobial agents [34]. For example, studies have used Pexiganan in combination with nisin to control the antimicrobial potential of planktonic and biofilm co-cultured diabetic foot ulcers and clinical strains [35]. And Pexiganan has been applied in the form of a cream as a wound dressing for mildly infected diabetic foot ulcers [2].This has further broadened their scope of application and increased their value, facilitating their gradual emergence as important candidate molecules for novel anti-infective drugs.

We selected four AMPs isolated from natural products for the study. Melittin, the main bioactive substance of bee venom, has excellent anti-inflammatory effects. Melittin, a basic peptide consisting of 26 amino acids, has the potential to penetrate cellular peptides not only as a drug but also as a therapeutic agent. [36]. Pexiganan is an orally active, broad-spectrum AMP derived from magainin [35]. Plectasin is the first fungal defensin-like AMP isolated by Mygind et al. in 2005 from the saprophytic ascomycete *Pseudoplectania nigrella*, which grows in Nordic pine forests [37]. Plectasin has exhibited significant antimicrobial activity against Gram-positive bacteria, especially *Streptococcus pneumoniae* and *Staphylococcus aureus*, as well as several strains that are resistant to conventional antibiotics [38]. In addition, our previous research identified cathelicidin-DM. It has broad-spectrum antimicrobial action. AMPs are novel antimicrobial molecules that are of great interest because they are considered promising candidates for further research and development, but the therapeutic mechanism of AMPs on bacterial vaginitis has not yet been elucidated by existing studies, so their efficacy in the treatment of bacterial vaginitis remains to be systematically evaluated. This study performed an evaluation of the *in vitro* anti-vaginitis activity of synthesized AMPs Pexiganan, plectasin, melittin, and cathelicidin-DM against Gram-negative and Gram-positive bacteria. Furthermore, we established an *in vivo* mixed infection model of *S. aureus* and *Escherichia coli* in the vaginas of mice to determine which AMPs play a therapeutic role in BV. The findings can be applied clinically at a later stage and provide an experimental basis for the proactive immunoprophylaxis of bacterial vaginitis.

## 2 Materials and methods

### 2.1 Materials

Agar and yeast extract were purchased from Biotopped. Tryptone was purchased from Angel Yeast Co. Sodium chloride was purchased from Tianjin Chemical Reagent No.3 Factory. MH medium was purchased from Beijing Aoboxing Biotechnology Co. DMEM was purchased from Gibco. Embryonic bovine serum and the CCK-8 kit were purchased from Beijing All Style Gold Biotechnology Co. IL-6 and IL-8 detection kits were purchased from Chern Technology Co.

### 2.2 Animals and strains

Twenty SPF Kunming (KM) mice (female, 5–12 weeks old, body weight of 20 ± 2 g) were purchased from the Yunnan University Laboratory Animal Center (License No. SCXK (Yunnan) K2021-0001).

The experimental strains were as follows: Gram-negative standard strain *Escherichia coli* ATCC25922, E*scherichia coli* ATCC35218, *Klebsiella pneumoniae* ATCC 700603, and the Gram-positive bacterium *Staphylococcus aureus* ATCC25923, all of which were purchased from the First People’s Hospital of Yunnan Province.


**Ethical approval:** The research related to animal use has been complied with all the relevant national regulations and institutional policies for the care and use of animals, and has been approved by the Animal Ethical Review Committee of Kunming University of Science and Technology (License No. SYXK (Yunnan) K2018-0008).

### 2.3 Peptide synthesis and purification

Four AMPs were synthesized, including three peptides with a high potential for clinical application (Pexiganan, plectasin, and melittin). Cathelicidin-DM AMPs were sourced from *Duttaphrynus melanostictus.* and purified by high performance liquid chromatography. The purity of all peptides exceeded 95% (Table 1).

**Table 1:** Peptide sequences used in this study

Peptide	Sequences (C′–N′)
Melittin	GIGAVLKVLTTGLPALISWIKRKRQQ
Plectasin	GFGCNGPWDEDDMOCHNHCKSIKGYKGGYCAKGGFVCKCY
Pexiganan	GIGKELKKAKKEGKAFVKILKK

### 2.4 Cell and bacterial culture

HepG 2 cells were removed from liquid nitrogen and quickly melted in a 37°C water bath for approximately 1 min. When some ice remained in the frozen storage tube, the cells were centrifuged at 1,000 rpm for 5 min. The storage liquid was discarded before the cells were gently mixed with 1 mL of complete medium, transferred into T25 bottles, filled to 3 mL with complete medium, and cultured in a thermostatic incubator (37°C, 5% CO_2_, 70–80% humidity). After the cells were attached, the fluid was replaced to continue the culture. The cell medium was laced in a T25 bottle and added with 1 mL of 0.25% trypsin (containing 0.05% EDTA). When most cells were rounded, DMEM was added to blow the cells, which were then transferred to a cryopreservation tube, centrifuged at 1,000 rpm for 5 min, discarded the supernatant, added with 1 mL of frozen storage liquid, mixed well in a cryopreservation box, placed in a −80°C refrigerator overnight, and finally placed in a liquid nitrogen tank.

The three strains *Staphylococcus aureus*, *Escherichia coli*, and *K. pneumoniae* were activated, inoculated in solid medium by plate streaking, and then placed inverted in a 37°C incubator until colonies grew. Single colonies were then picked and transferred to MH medium until they reached logarithmic growth.

### 2.5 Evaluation of *in vitro* activity of AMPs

#### 2.5.1 Minimum inhibitory concentration (MIC)

Using the two-fold dilution method, the OD_600_ of the bacterial solution was detected by the turbidimetric method or on a UV photometer. The bacterial solution was diluted to 1.0 × 10^6^ CFU/mL with liquid MH medium according to 1OD_600_ = 1 × 10^9^ CFU/mL. Pre-fill a sterile 96-well plate with 100 μL of sterile water. Then, 174.4 μL of sterile water and 25.6 μL of the AMPs were added to the first well, which were then mixed and sequentially diluted. 100 μL was withdrawn from well 10 and discarded. Then, 100 μL of bacterial solution diluted to a certain concentration was added into each well. The cells were then cultivated in a 37°C constant temperature incubator for 16–18 h. The AMP concentrations of each well were 128, 64, 32, 16, 8, and 4 μg/mL. A blank control was set up to contain only the bacterial solution. The negative control contained blank medium only. The wells were observed for the presence or absence of bacterial growth and the lowest antimicrobial concentration without bacterial growth was the MIC of the antimicrobial peptide for that bacterium.

#### 2.5.2 Cytotoxicity assay

The inhibitory effect of the AMPs on HepG2 cells was detected by the CCK-8 method, whereby a higher absorbance value corresponded to higher cell activity. Cells and culture solution were taken from culture flasks containing DMEM and placed in a test tube, shaken well, poured into a flat dish, and then aspirated. Then, 100 μL of cell and culture solution mixture was added to each well of a 96-well plate, after which, 5 × 10^3^ cells were added per well to undergo pre-culture at 37°C in a cell culture box for 12–24 h. The peptides were sequentially added to cell-lined 96-well plates at concentrations of 128, 64, 32, 16, 8, and 4 μg/mL and were then incubated in a cell culture incubator for 24 and 48 h. The medium was then discarded, 500 mL of 1× PBS was added for washing, basal medium was added, cell growth status was micrographically visualized, and the cells were photographed. Each well was protected from light by adding 10 μL of CCK-8 solution and the plate was incubated in a cell culture incubator for 2 h. Absorbance values at 450 nm were measured using an enzyme marker. The cell activity (%) was calculated by the formula

\[{[}{A}_{\text{experiment}}-{A}_{\text{blank}}]/{[}{A}_{\text{control}}-{A}_{\text{blank}}]\times 100 \% .]\]

### 2.6 BV model

All experimental mice were subjected to a drug sham treatment by the subcutaneous injection of estradiol benzoate (0.5 mL/10 g) in the groin once every other day for three injections in total. On day 6, after observing that the vaginal opening of the mice was significantly dilated, the mice were allowed to proceed to the next stage of modeling.

We prepared 1 × 10^6^ CFU/mL of a mixed *Staphylococcus aureus* and *Escherichia coli* bacterial solution, used a dropper to aspirate 0.25 mL of each mixed bacterial suspension, slowly inserted this into the vagina of each mouse at about 1–1.5 cm deep, released the bacterial solution into the vagina, slowly withdrew the syringe outward, used sterile tweezers to clamp a sterile medical cotton ball dipped in the corresponding bacterial suspension to block the vaginal opening, and then held the position of head-down and feet-up for at least 2–3 min; this operation was repeated once a day for a total of 4 days. The secretions of each group were taken the day after the inoculation and on the seventh day for microscopic examination and bacterial culture, respectively, and photographs were also taken.

### 2.7 Animal group and treatment

Sixteen mice with successful modeling were taken. They were randomly divided into four groups of four mice each. Group A was the model group, group B was treated with melittin, group C was treated with Pexiganan, and group D was treated with Cathelicidin-DM. Another four normal mice were taken as the blank (normal) control group. The model group and the blank control group were washed with warm saline, while the experimental group was treated with 16 μg/mL of AMPs for 7 consecutive days. Mouse vaginal lavage fluid was collected for culture and microscopy, respectively.

### 2.8 Blood collection and detection of inflammatory factors

Blood was collected from the tail vein of each mouse, placed at room temperature for approximately 1 h, centrifuged, and the supernatant was collected for spare. The blood was then assayed by the enzyme-linked immunosorbent assay (ELISA) to detect the expression of localized Th1 and Th2 cell-specific immunity factors (IL-8 and IL-4) in the vaginas of the BV model mice.

### 2.9 Statistical processing

All data were processed with the software Prism 8 and the results were plotted. Experimental results were expressed as mean value ± standard deviation (SD) (*n* = 3) using the *t*-test between groups. *P* < 0.05 was considered statistically significant.

## 3 Results and discussion

### 3.1 MIC

The inhibitory activities of the four peptides were detected by using the multiplicative dilution method. The MIC results showed that the three peptides melittin, Pexiganan, and cathelicidin-DM had good inhibitory activities against both Gram-negative and Gram-positive bacteria, and also exhibited broad-spectrum antimicrobial activities, as shown in Table 2. The most significant inhibitory effect was observed for Pexiganan, for which the lowest MIC was 8 μg/mL against Gram-positive and Gram-negative bacteria. Our results indicate that plectasin did not significantly inhibit Gram-negative and Gram-positive bacteria at low concentrations; however, it has previously been shown to have significant antibacterial activity against Gram-positive bacteria, especially *Streptococcus pneumoniae* and *Staphylococcus aureus* [39]. Therefore, whether the peptide has bacteriostatic activity at high concentrations needs to be further investigated, which is the basis for the subsequent application of the peptide. The value of these three peptides featuring good antimicrobial activity was further evaluated for clinical application by determining the effect of the cell viability of these peptides.

**Table 2:** Summary of MIC for experimental bacteria (μg/mL)

Bacterial name	Melittin	Pexiganan	Cathelicidin-DM	Plectasin
Strain *Escherichia coli* ATCC25922	16	8	32	>128
*Escherichia coli* ATCC35218	32	7	128	>128
*Staphylococcus aureus* ATCC25923	8	8	32	>128
*Klebsiella pneumoniae* ATCC 700603	64	16	128	>128

### 3.2 Cytotoxicity assay

**Figure 1:**
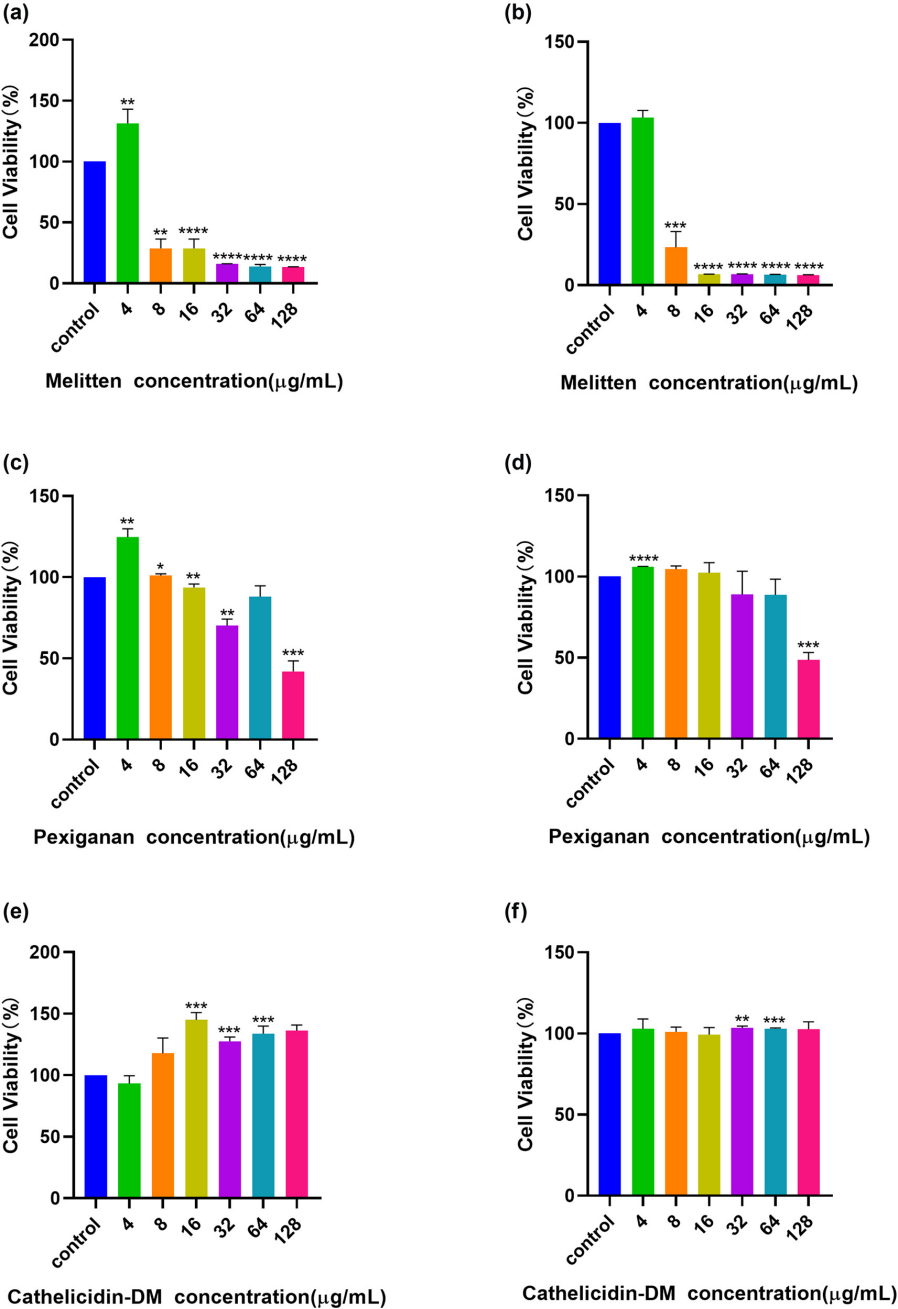
Effect of AMPs on the cell activity of HepG 2 cells (a), (c), and (e) after 24 h; (b), (d), and (f) after 48 h. P* < 0.05, P** < 0.02, P*** < 0.0002, P**** < 0.0001.

The three studied peptides that exhibited good antibacterial activity (melittin, Pexiganan, and cathelicidin-DM) were selected by fold-ratio dilution. The potential of these three peptides for clinical application was subsequently assessed by measuring their effect on cell viability. The inhibitory effects of melittin, Pexiganan, and cathelicidin-DM on HepG2 cells were detected by the CCK-8 method, with higher absorbance values representing higher cell activity. As shown in Figure 1, it can be seen that the melittin was highly toxic to the cells, such that the survival of the cells gradually decreased as the peptide concentration increased. Melittin caused cytotoxic effects leading to necrosis and apoptotic cell death. In order to overcome this problem, subsequent research should explore alternatives. As the concentration of the peptide increased, the cell survival rate was significantly reduced compared to the control. This indicates that the peptide has some toxicity. Cathelicidin-DM was not toxic at low concentrations, and there was no significant change in cell survival compared to control as the peptide concentration increased, suggesting that the peptide also had little effect on cell activity.

### 3.3 Establishment of the animal model

**Figure 2:**
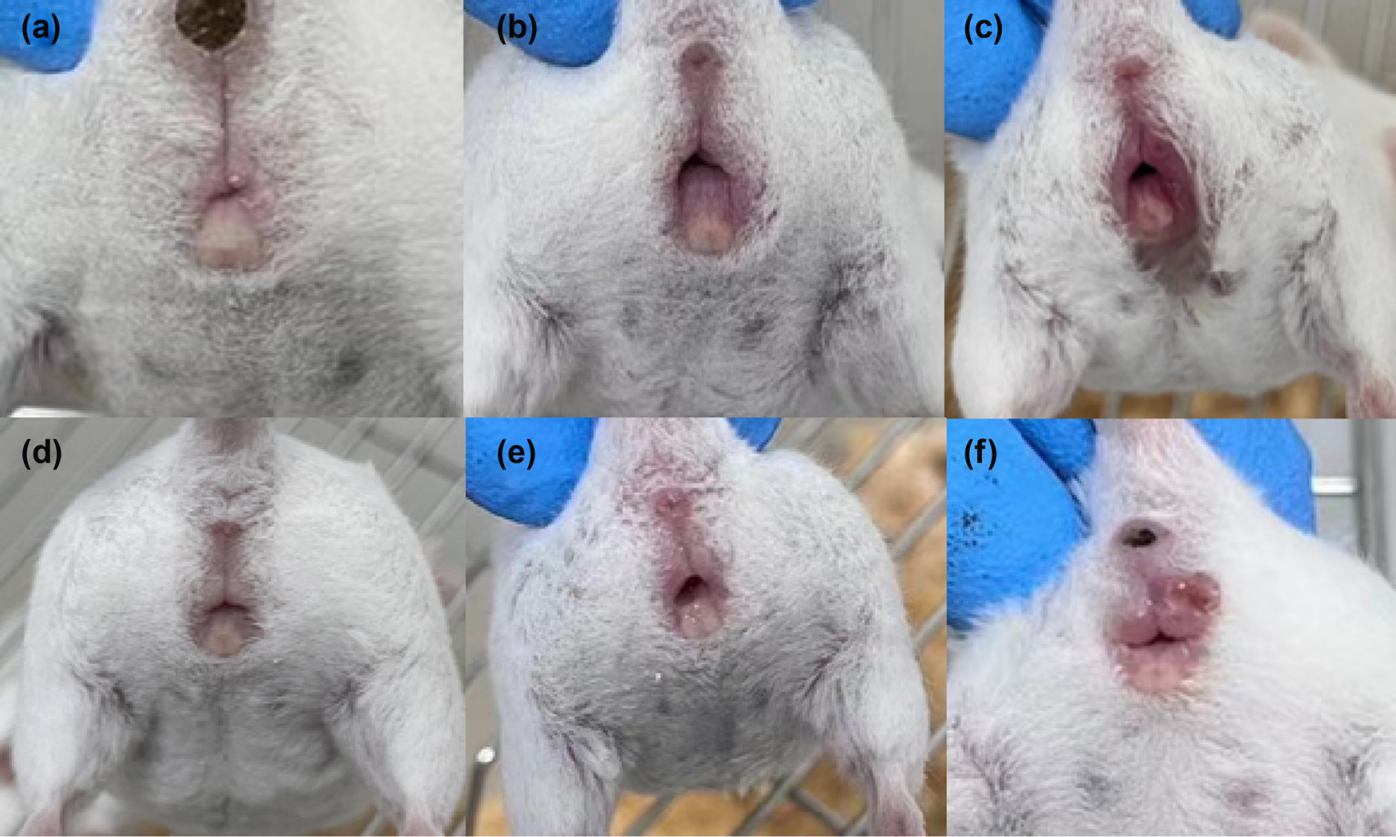
The mouse vagina 2 weeks after the infusion of bacterial fluid (a) and (d) blank controls without any treatment; and (b), (c), (e), and (f) the vaginal condition of mice after the infusion of bacteria.

To further assess their potential as topical antibacterial agents, we evaluated the *in vivo* bacteriostatic effects of the three peptides melittin, Pexiganan, and cathelicidin-DM against *Staphylococcus aureus*- and *Escherichia coli*-induced vaginitis. First, a mixture of *Escherichia coli* and *Staphylococcus aureus* was instilled into the mouse vagina to establish a mouse bacterial vaginitis model. As shown in Figure 2, the vaginal appearance of mice in the blank group showed no congestion, redness, or swelling, and the vaginal secretions were homogeneous, with no obvious purulent, bloody, or abnormal secretions. The vaginas of mice in Groups C and D were slightly red and swollen, while the vaginas of mice in Groups E and F were red and swollen with localized ulceration. The results showed that the BV model was successfully established.

### 3.4 Bacterial culture and microscopy

**Figure 3:**
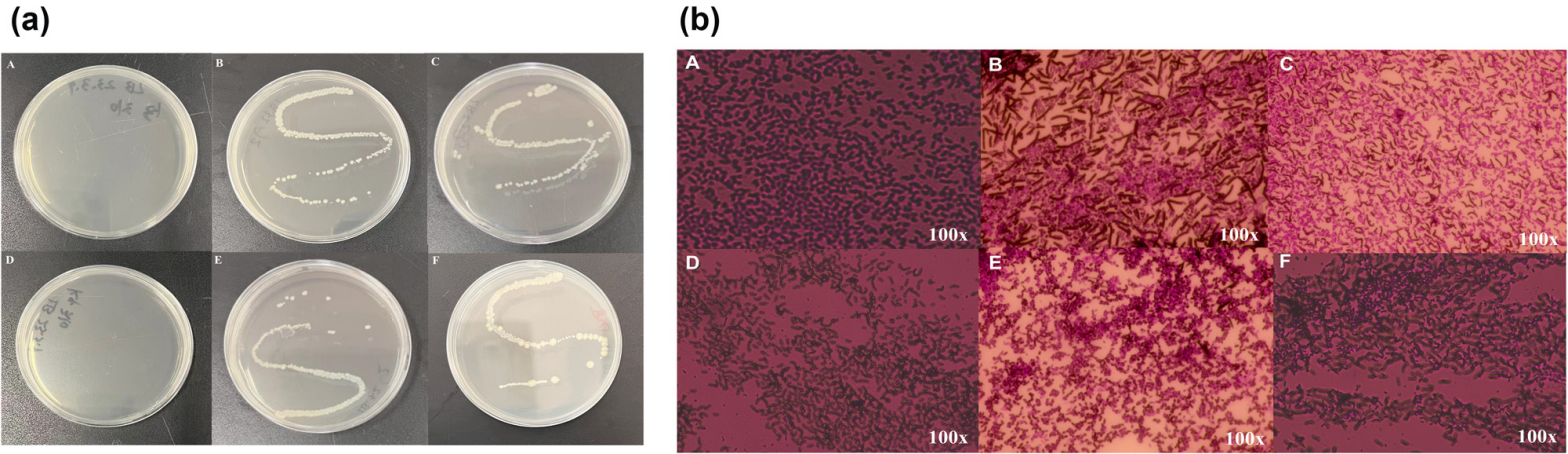
Bacterial culture and microscopy: (a) Bacterial culture; and (b) Gram stain: (A) and (D) blank controls without any treatment; and (B), (C), (E), and (F) the vaginal condition of mice after the infusion of bacteria.

To further validate that the bacterial vaginitis model had been successfully established, vaginal lavage fluid was collected vaginally for culture. As shown in Figure 3a, the extensive growth of colonies can be observed all over the plate. The cultured bacteria were then subjected to Gram staining. As shown in Figure 3b, Gram-positive and Gram-negative bacteria were observed in the colonies. Morphologically, *E. coli* and *Staphylococcus aureus* were clearly observed. From vulvar observations and microscopic examination, it was hypothesized that the BV model was successfully established, and could hence undergo experimental treatment.

### 3.5 Grouping and treatment

**Figure 4:**
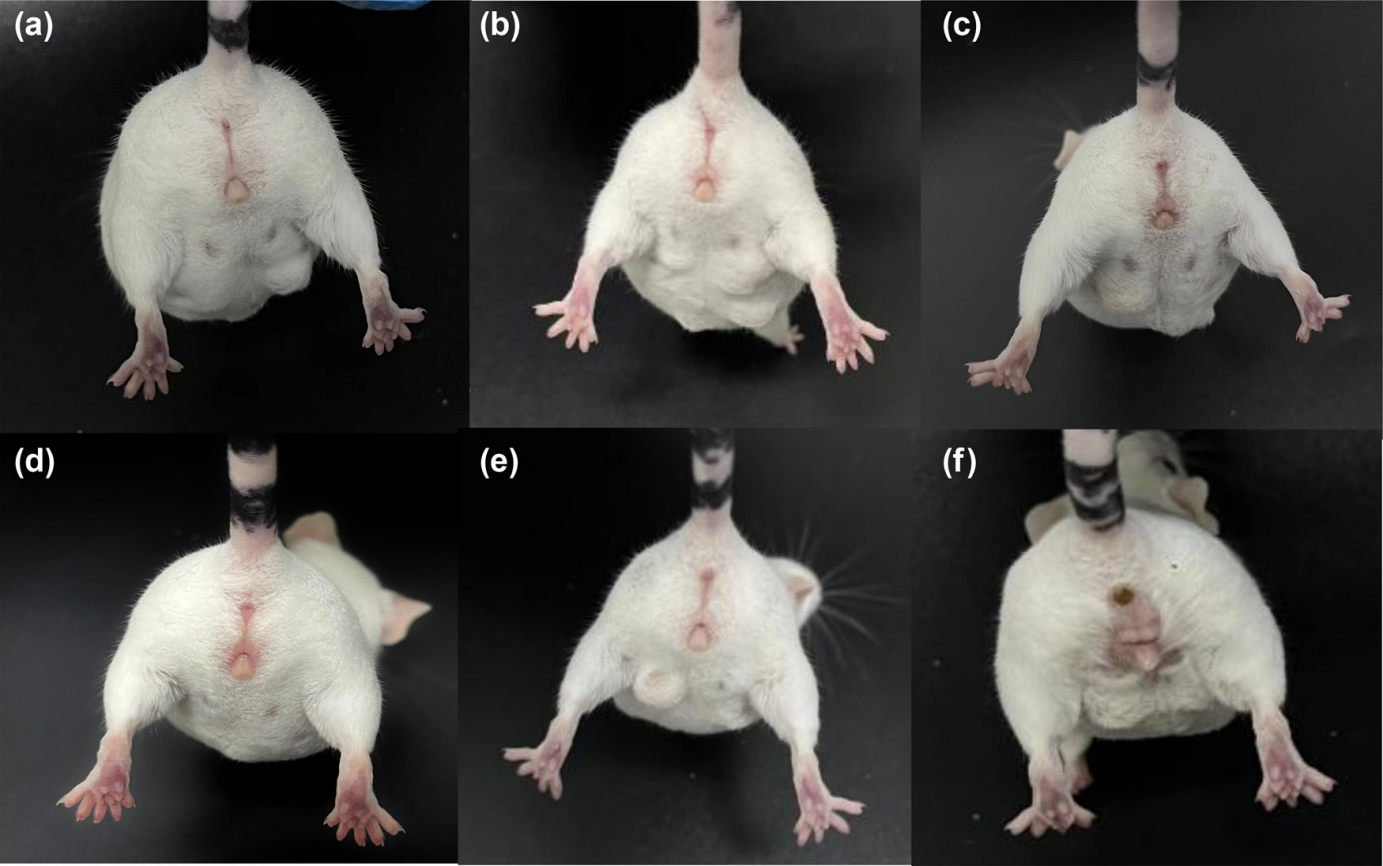
Recovery of the vulva in mice after treatment (a) and (d) blank controls without any treatment; and (b), (c), (e), and (f) drug treatment group.

**Figure 5:**
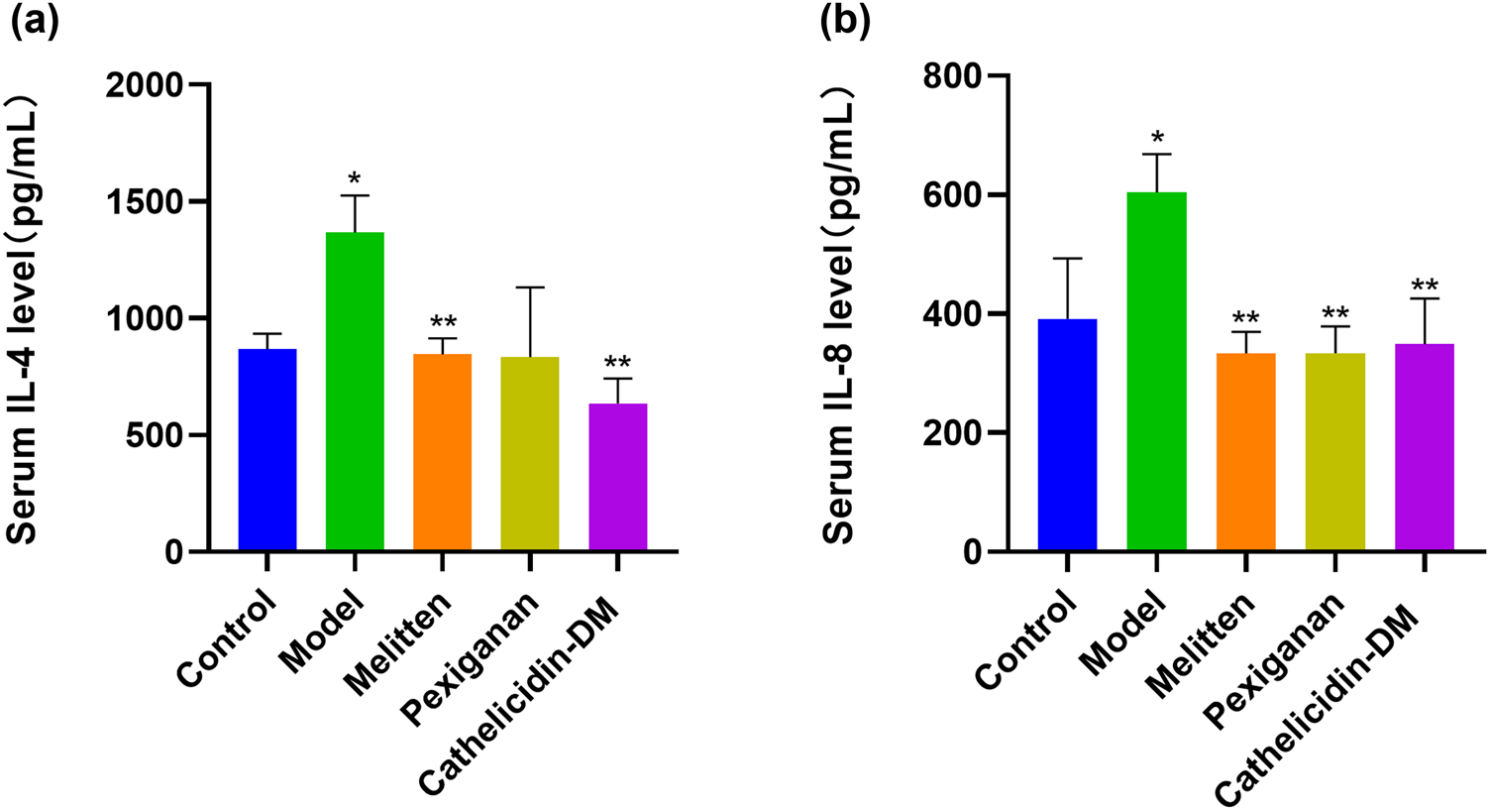
Inflammatory factors in the serum of vaginitis model mice in different treatment groups. (a) IL-8 concentration in the serum of vaginitis model mice in different treatment groups; and (b) IL-4 concentration in the serum of vaginitis model mice in different treatment groups. P* < 0.05, P** < 0.02.

As shown in Figure 4, after the administration of the AMPs, the vulvar erythema status of the mice in the treatment group was significantly improved compared to that of the model group, indicating that all three AMPs have potential therapeutic effects on bacterial vaginitis. ELISA was then conducted to detect the expression of localized Th1 and Th2 cell-specific immune factors (IL-8 and IL-4) in the vaginas of the BV model mice. As shown in Figure 5, after administration, the content of IL-4 in the treatment group was significantly reduced compared to that measured in the model group, indicating that all three AMPs have potential therapeutic effects on bacterial vaginitis. After the administration of the AMPs, IL-8 levels were also significantly reduced in the treatment group compared to the model group. These results showed that the three AMPs also had good bacteriostatic effects *in vivo*. In conclusion, these three AMPs exhibited good bacteriostatic activity both *in vivo* and *in vitro*, thus having potential therapeutic effects on bacterial vaginitis.

## 4 Conclusion

In this study, the three AMPs Pexiganan, melittin, and cathelicidin-DM, which play a therapeutic role in bacterial vaginitis, were evaluated for their *in vitro* antivaginitis activity and assessed for their inhibitory activity in a mouse model of Gram-negative and Gram-positive bacterial infection. Among them, plectasin at a concentration of 128 μg/mL did not inhibit either Gram-negative or Gram-positive bacteria. Melittin had an antibacterial effect both *in vitro* and *in vivo*, but had a greater effect on HepG2 cell activity compared to Pexiganan and cathelicidin-DM, so whether it can be applied to the clinical treatment of vaginitis needs to be further investigated. Cathelicidin-DM exhibited antimicrobial activity both *in vivo* and *in vitro* and had no great effect on HepG2 cell activity. Therefore, the results suggest that Pexiganan and cathelicidin-DM have great potential for clinical application in the treatment of vaginitis.
